# Echoes of the Month

**Published:** 1923-09

**Authors:** 


### ECHOES OF THE MONTH.

### THE COST OF SMALLPOX.

Remarkable figures showing the decline of smallpox in London during recent years and the heavy cost of treating the disease in the hospitals, were quoted by Dr. W. McConnell Wanklyn, principal Assistant Medical Officer of Health to the London County Council, in a lecture at the London Hospital. In 1921, the lecturer stated, there were 336 cases of smallpox in the country at large, and two cases in London; in 1922, there were 974 cases in the country, and 65 in London; and in the present year there had so far been about fifteen hundred cases in the country and none in London. Dealing with smallpox prevention from the point of view of finance, he mentioned a case in which a single patient had cost the local authority as much as £500. The recent epidemic in Gloucester was stated to have cost about £200 a case. For several years before 1904 the number of cases in London averaged about 700 annually. In that year, therefore, it could quite reasonably have been anticipated that in the following twenty years the aggregate number of cases would be about 14,000. As yet, however, there had only been 340 cases since 1904, with a saving, at £200 a case, of £2,800,000, in the cost of treatment. This very satisfactory result he attributed to improved methods of diagnosis, which enabled smallpox to be " spotted " as soon as it arrived, and to the splendid " team work " of the London medical profession.

### "A RACE TO DEATH."

The decline in the birth-rate—" the evil which is killing France "—has now been set forth for the study of Frenchmen in a pamphlet entitled " The Death or Life of France," for which M. Paul Naury, its author, has just been awarded the Michelin Natality Prize. In 1920 there was a shrinkage of 21,000 in the birth returns, while in the following year births had still further diminished by 53,000. The marriage-rate is following the same downward course. Of the 86 departments of France, 64 are now more sparsely populated than they were sixty years ago. While the population of Germany has increased by 21,000,000 during the last half-century, that of France has risen by only 3,000,000. England in this period has increased her population by 16,000,000, Italy by 13,000,000, and Spain by 4,500,000. In no other country has the rate of depopulation approached that of France. " It is a race to death, the suicide of a great people," says M. Paul Naury. " Only a mighty effort, transforming both our laws and manners, can save us now. This effort must be made immediately, or France is lost."

### A CHAMBER OF HORRORS.

In spite of " line upon line and precept upon precept," says *The Modern Hospital*, the destruction of valuable hospital equipment through careless handling is a source of great concern to the average hospital superintendent. One superintendent of nurses has a " chamber of horrors," which the students in the training school are invited to inspect occasionally. It contains such articles as rubber catheters burnt during sterilisations, record syringes with piston impacted, the serum having been allowed to dry, patients' clothing badly creased through careless folding, stained linen after boiling in laundry, clinical thermometers broken in a single week, a scrubbing brush which was responsible for the obstruction of sewer, etc. This visual method has been found to be impressive to the average student.

### AN AMERICAN HOSPITAL MENU.

The traditional diet of the hospital is a thing of the past so far as the Harper Hospital at Detroit is concerned (says the *Harper Hospital Bulletin*). A day's menu, taken at random, as a sample, is appended. A graduate dietitian furnishes the scientific knowledge of food and a chef who has officiated at good hotels directs the preparation of the meals. Buying the best of foods, with the best of planning and cooking, our patients have good meals whenever the hard-hearted physicians allow them real food:—Menu.—*Breakfast.*—Fruits: Oranges, St. Prunes. Cereals: Rolled Oats, Puffed Rice. Bacon or Eggs. Bread; Toast; Wheat; Whole Wheat; Graham Bread. Coffee; Tea; Milk. *Dinner.*—Essence of Tomato, Celery and Olives. Chicken à la King. Boiled Rice; Baked Potatoes; Buttered String Beans; Creamed Peas. Pear and Cottage Cheese Salad. Head Lettuce. Ice Cream. Tea or Milk. *Supper.*—Cream Reine Margot. Ralstons. Broiled Ham. Cr. Tunafish with Peas on Toast. Baked Potatoes. Head Lettuce with 1,000 Island Dressing. Royal Anne Cherries. Vanilla Wafers. Tea or Milk.

### HEALTH OF THE AMERICAN INDIAN.

The seventy-eight hospitals for Indians in the United States have treated over 20,000 Indians during the past year. These institutions have a total capacity of 2,400 beds. The Department of the Interior also operates a hospital exclusively for the treatment of nervous and mental diseases among Indians at Canton, South Dakota. The health of the Indian is one of the biggest problems which the Bureau of Indian Affairs has to meet, as was pointed out in the recent statement made by the Commissioner of Indian Affairs at Washington. The Indian population of the United States, he said, is 340,197, of whom 225,000 are under the direct supervision of the Bureau of Indian Affairs of the Interior Department. Of this number approximately 25,000 Indians are afflicted with some form of tuberculosis and approximately 80,000 have trachoma and other eye diseases. These institutions not only furnish treatment, but, in addition, prepare to give a modified educational course to patients.

### THE CHILD AND ITS HANDKERCHIEF.

" The most important article of baby's clothing is his pocket handkerchief; if he uses a handkerchief from his birth he will avoid most of his troubles in life." This insistence upon the handkerchief is part of the medical creed—as far as children are concerned—of Dr. Octavia Lewin, medical officer of the Soho, Strand, and Malfair Maternity and Child Welfare Centres of the City of Westminster Health Society. Every child, says Dr. Lewin, should have a handkerchief fastened by a button to the left breast pocket. This teaches it to bow the head when it blows its nose—which is the correct attitude.

### EPIDEMICS PREVENTED BY THE LEAGUE.

The outbreak of a serious epidemic was recently prevented by the Health Organization of the League of Nations arranging for the vaccination of 720,000 of the refugees in Greece. The task was completed at a cost of £5,000, or at the rate of 1¾d. a head. The campaign against typhus in Poland is still being conducted with vigour. There are now fifty-eight fully-equipped isolation hospitals, each bearing a marble slab inscribed: " Built by the Polish Government and the League of Nations." There is such general confidence in the efficiency of the League's health organisation that Governments are prompt and eager in supplying all necessary information. One result of this ready co-operation is that during the past three months the League has been able to prevent the outbreak of three threatened attacks of plague in various parts of Eastern Europe.

### WHERE THE SUN NEVER RISES.

Lancaster Town Council have decided to apply to the Ministry of Health for an order declaring certain slum property in St. Leonardgate as an "unhealthy area." The area comprises 3,429 square yards, and includes 56 houses in the occupation of 251 people. Over the past ten years the death-rate in this area was 108 per cent. above that of the rest of the borough, infantile mortality was 11 per cent. up, and deaths from respiratory diseases were 556 per cent. above the rest of the borough. The birth-rate was also 80 per cent. above that of the borough. Here the sun never rises, and there is a minimum of fresh air.

### A MEDICAL STUDENTS' TOUR.

A party of eighteen British medical students is starting on a three weeks' tour of the principal European hospitals. They will visit the most famous clinics in France, Belgium, Holland, Germany, Czecho-Slovakia, Austria, and Switzerland, and see for themselves the latest developments of research and of medical and surgical practice abroad. The tour has been arranged by the National Union of Students, and in each country the party will be met and taken in charge by the medical students on the spot, who will look after their English guests and assist the authorities of the hospitals and pathological institutes to show them everything of interest and educational value.

### WHITE BREAD MORE DEADLY THAN STARVATION.

Speaking at a meeting between representatives of the Trade Union Congress General Council and the Medical Council of the People's League of Health, Sir Harry Baldwin said that every disease in the medical directory could be caused through the neglect of the teeth, and one of the commonest causes of bad teeth is incorrect diet. This particularly affected the working classes, as in poor families about two-thirds of the food is made up of bread and flour—that is, white bread, from which all the more valuable constituents have been removed. A tax ought to be put upon white bread and applied to make wholemeal bread cheaper. Not only is white bread useless as a food, but it is actually harmful. Pigeons fed on white bread and water died sooner than those that were given nothing.

### LIFE-SAVING BY TELEGRAM.

The telegraphic code words, "positive" and "negative," used for describing the results of medical tests of swabs from suspected cases of diphtheria, have been scrapped by the Post Office authorities. The words "bacilli not found" or "bacilli present" have been substituted. Telegraphic confusion of the words "positive" and "negative" was not infrequent.

### THE CRICKETS ON THE HEARTH.

According to the *Daily Express*, Swindon, the "railway town," is swarming with crickets. There are hundreds of thousands of them. They troop into the houses in regiments. They go up the stairs and into the bedrooms. All night there is one monotonous, irritating, sleep-destroying chirrup. No one can sleep. To make matters worse, fleas arrive like cavalry on the backs of the crickets, and bite while their allies chirrup. As fast as the crickets are slaughtered, fresh legions arrive. One man showed the *Express* correspondent a box containing many hundreds of the enemy—one night's "bag." "The floor of a bedroom I visited was strewn with the dead. The family, unable to sleep, had spent the night in 'swatting' crickets." They measure from one inch to two inches in length. It is supposed that the plague originated in a refuse tip, where the crickets, conveyed there from a bakery, bred quickly in the heat.

### WATER-CRESS AND LETTUCE.

The old type of Cockney was devoted to water-cress ("water-creeses" he called it), but in London now, for some mysterious reason, it is not eaten nearly so much as it used to be, and the practice of eating fresh lettuces, which is of the highest dietetic value, is also said to be declining. One theory is that water-cress fell off in popularity as the result of a scare many years ago associating water-grown vegetables with typhoid fever, but there is no such danger when the cress comes from pure country streams. One excellent use of water-cress is to eat it with cheese.

### SEEING BACTERIA.

Dr. Shore, Lecturer in Biology, at St. Bartholomew's Hospital, has retired after 40 years' service. Dr. Shore has accumulated a fund of good stories during his long experience. One relates to a student who was "an awful dunce." After many failures "we at last," says the doctor, "managed to teach him that there were two sorts of blood-corpuscles. Then we asked him what they were. He replied 'Male and female.' On another occasion the same student was asked to state what was under a certain microscope. He replied 'Bacteria,' and was correct. We asked him how he knew. He said: 'Well, if I look down a microscope and see nothing at all I always say, bacteria.'"

